# Tuberculosis among Patients Undergoing Solid Organ Transplantation or Dialysis in a Low-Endemic Country, 2004-2017

**DOI:** 10.1155/2020/7636975

**Published:** 2020-04-25

**Authors:** Marie Helleberg, Daniel Cho, Christina Ekenberg, Søren Sørensen, Marianne Rix, Finn Gustafsson, Allan Rasmussen, Michael Perch, Peter H. S. Andersen, Jens D. Lundgren, Aase Bengaard Andersen

**Affiliations:** ^1^Department of Infectious Diseases, Copenhagen University Hospital, Rigshospitalet, Blegdamsvej 9, 2100 Copenhagen Ö, Denmark; ^2^Centre of Excellence for Health, Immunity and Infections (CHIP), Copenhagen University Hospital/Rigshospitalet, Blegdamsvej 9, 2100 Copenhagen Ö, Denmark; ^3^Department of Nephrology, Copenhagen University Hospital, Rigshospitalet, Blegdamsvej 9, 2100 Copenhagen Ö, Denmark; ^4^Department of Cardiology, Copenhagen University Hospital, Rigshospitalet, Blegdamsvej 9, 2100 Copenhagen Ö, Denmark; ^5^Department of Surgery C, Copenhagen University Hospital, Rigshospitalet, Blegdamsvej 9, 2100 Copenhagen Ö, Denmark; ^6^Department of Infection Epidemiology and Prevention, The State Serum Institute, Artillerivej 5, 2300 Copenhagen S, Denmark

## Abstract

**Background:**

The risk of active TB among solid organ transplant (SOT) recipients and patients initiating chronic dialysis in a country with low incidence of TB is not well elucidated.

**Methods:**

Patients aged >18 years who were transplanted with a solid organ or initiated chronic dialysis at Copenhagen University Hospital in the period 2004-2017 were followed from date of transplantation or initiation of dialysis. Data on demographics and outcomes were obtained from nationwide registries.

**Results:**

We included 1,989 SOT recipients and 1,305 patients initiating chronic dialysis, who were followed for a total of 9,785 and 4,196 person-years (PY), respectively. Only a minority of patients had been screened for latent TB prior to SOT or initiation of dialysis. The incidence rates (IRs)/100,000 PY of TB among patients from medium/high TB endemic areas were 358 (95% CI 115-1,110) and 1,266 (95% CI 681-2354) for SOT and dialysis patients, respectively, whereas IRs among patients of Danish origin were 11 (95% CI 2-81) and 31 (95% CI 4-218).

**Conclusion:**

The incidence of TB among immunosuppressed immigrants from medium/high TB endemic countries was very high, while the risk of TB among patients from low-endemic countries was minimal.

## 1. Introduction

Compromised immune function increases the risk of developing active tuberculosis (TB). Apart from HIV, two important risk factors for TB reactivation are solid organ transplantation (SOT) and chronic renal failure requiring dialysis [1]. Progression to active TB is a serious condition in patients with impaired renal function and in SOT recipients. Extrapulmonary disease is common, symptoms and signs are often unspecific which causes difficulties in timely diagnosis, and rates of disseminated disease at time of diagnosis are high [2]. Treatment is a challenge due to drug-drug interactions with rifampicin and the various immunosuppressant drugs. Dosing is difficult in patients with renal impairment, and it is reported that >50% of patients on dialysis have significant side effects to anti-TB drugs [3].

WHO guidelines and the Infectious Diseases Society of America recommend screening of high-risk populations for latent TB infection and prescribe preventive therapy [4, 5].

Denmark, like most European and so-called western countries, has a low and declining incidence of TB. However, TB high-risk subpopulations exists, especially in people originating from high TB endemic areas like Africa, Asia, and Greenland. In a “globalized world,” large population groups migrate due to political instability to Europe and northern America, but the contact pattern to their country of origin often remain close with a high level of travelling activity also by the young 2nd-generation immigrants. Some patients may even present to the health system with a recently transplanted organ, acquired in a TB endemic country. The magnitude of the problem of TB reactivation in patients undergoing immunosuppressive therapy in a country with a low TB incidence is not well elucidated.

The aim of this study was to estimate the risk of developing active TB among SOT recipients and patients requiring chronic dialysis in a country with a low incidence of TB.

## 2. Methods

### 2.1. Setting

Denmark has a total population of 5.8 mil inhabitants, and 493.468 are immigrants or 1st-degree descendants from nonwestern countries as of 2018 [6]. In 2017, the incidence of TB in Denmark was 4.8 per 100,000 population, and 75% of TB cases were among immigrants [7].

“Rigshospitalet” is a third-level, referral university hospital located in Copenhagen, the capital of Denmark. The hospital serves as a national transplantation center for liver and lung transplantations and a regional center for kidney and heart transplantations.

### 2.2. Study Population

We included all consecutive patients, aged >18 years, who were transplanted with a solid organ or who initiated chronic dialysis at Rigshospitalet, Copenhagen University Hospital, in the period 2004-2017.

### 2.3. Data Sources

Data on ethnic origin, immigrations, emigrations, and dates of death were obtained from the Danish Civil Registration System. A local data warehouse, entitled “The PERSIMUNE data warehouse,” provided data on laboratory tests, dialysis, and date and type of SOT [8]. Nationwide data on laboratory tests are only complete from 2010 and onwards. We obtained nationwide data on diagnoses of active TB from the Danish National TB registry and from the Danish National Patient Registry [9].

Data were linked using the unique 10-digit civil registration number which is assigned to all people living in Denmark at birth or at immigration.

### 2.4. Definitions

Greenland and countries in Eastern Europe, Africa, Asia, and South America were categorized as medium/high TB endemic countries; and countries in Northern and Western Europe, Northern America, and West Pacific were categorized as low TB endemic countries, i.e., <10 cases per 100,000 population. Second-generation immigrants were categorized as of Danish origin in the analyses as they were too few to be included as a separate category.

### 2.5. Statistics

Patients were followed from date of SOT or date of initiation of chronic dialysis until date of diagnosis of active TB, death, emigration, or 28 February 2017, whichever came first. Patients who initiated dialyses and later received a SOT were censored at the time of SOT and were included in the group of SOT patients at the date of transplantation. Patients who received a SOT and later initiated chronic dialysis accrued follow-up time in the SOT group irrespective of dialysis.

Time to development of active TB was estimated using the Kaplan-Meier methods. Relative risks were estimated by the multivariate Poisson regression analyses. Variables included in the multivariate analyses were gender, age, calendar year of SOT/start of dialysis, and origin. There were no missing data for these variables in the dataset.

Stata, version 15.1 (StataCorp, College Station, Texas, USA), was used for data analyses.

The study was approved by the Danish Patient Safety Authority I-Suite no: 03605.

## 3. Results

### 3.1. Baseline Characteristics

We included 1,989 SOT recipients and 1,305 patients who initiated chronic dialysis at Copenhagen University Hospital, Rigshospitalet, in the period 2004-2017. A total of 225 of the SOT recipients had been on chronic dialyses at Rigshospitalet prior to transplantation. Baseline characteristics are summarized in Table 1. Of the 1,989 SOT recipients, 917 (46.1%) had a kidney transplant, 530 (26.7%) a liver transplant, 365 (18.5%) a lung transplant, 158 (7.9%) a heart transplant, and 15 (0.8%) a kidney/pancreas or kidney/liver transplant. The immunosuppressive regimens used at the center in the study period are described in the supplemental material (available here). Most SOT recipients, 1,805 (91.0%), were from low TB endemic countries, but 184 (9.0%) originated from a medium/high TB endemic country. Of patients who initiated chronic dialysis, 1,092 (83.7%) were from low TB endemic countries and 213 (16.3%) were from a medium/high TB endemic country. A total of 43 SOT and 16 dialyses patients were second-generation immigrants from medium/high TB endemic countries.

Thirteen SOT recipients and 16 dialysis patients had been treated for active tuberculosis prior to SOT or start of chronic dialysis. None of the patients who were on chronic dialysis prior to SOT had been diagnosed with TB prior to transplantation.

### 3.2. Incidence of Active TB after SOT/Dialysis

SOT recipients were followed for a total of 9,785 person-years (PY) of follow-up, median 4.4 years (interquartile range (IQR) 1.7-7.4), and dialysis patients were followed for a total of 4,196 PY, median 2.4 years (IQR 1.1-4.6). Four cases of active TB were diagnosed in the SOT patient group (3 cases in kidney transplant recipients and 1 liver transplant recipient) and eleven cases among the patients on dialysis.

The incidence rates (IRs)/100,000 PY of active TB were 41 (95% confidence interval (CI) 15-109) and 262 (95% CI 145-473) for SOT and dialysis patients, respectively (Table 2), which is 8.5 and 55 times higher than in the general population, respectively. For kidney and non-kidney transplant recipients, the IRs/100,000 PY of active TB were 108 (95% CI 35-336) and 17 (95% CI 2-119), respectively. Three of the four TB cases among SOT recipients were patients from medium/high TB endemic countries and one was of Danish origin. Ten of the patients who developed active TB after initiating chronic dialyses were from TB endemic countries and one was of Danish origin. There were no cases of TB among second-generation immigrants. The IRs/100,000 PY of TB among patients from medium/high TB endemic areas were 358 (95% CI 115-1,110) and 1,266 (95% CI 681-2,354) for SOT and dialysis patients, respectively.

**Figure 1:**
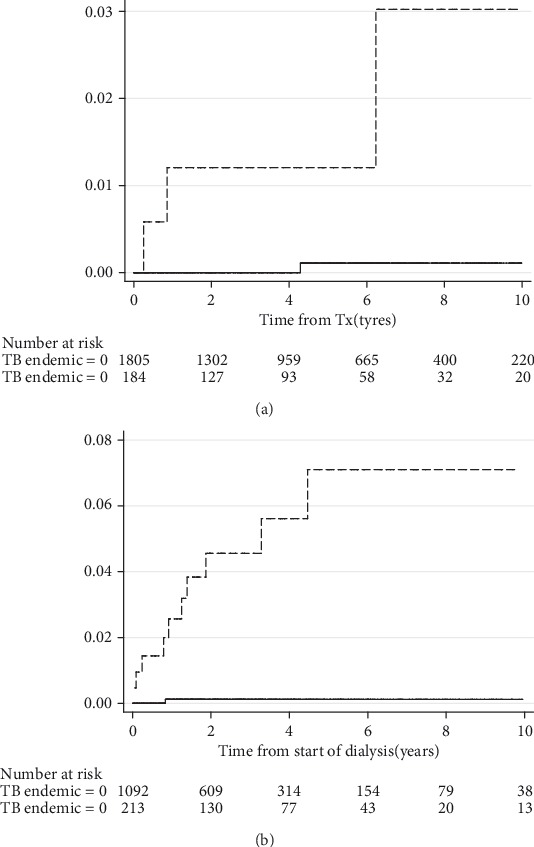
Time to diagnosis of active tuberculosis among solid organ transplant recipients (a) and dialysis patients (b) born in medium/high TB endemic countries (broken line) versus low TB endemic countries (full line).

The Kaplan-Meier curves of time from transplantation or start of chronic dialysis to a diagnosis of active TB are shown in Figure 1. The median time to TB diagnosis was 2.6 years (IQR 0.6-5.3) and 0.9 years (IQR 0.2-1.9) among SOT and dialysis patients, respectively. No cases of TB were diagnosed within 3 months of transplantation, but two cases (50%) were diagnosed within one year of SOT, whereas three (27%) and six (34%) patients were diagnosed with active TB within three months and one year after starting chronic dialysis, respectively.

Female gender and origin from Greenland or another medium/high TB endemic area were risk factors for being diagnosed with active TB (Table 2).

### 3.3. TB Diagnoses

Of the fifteen patients who developed active TB, ten were diagnosed with pulmonary TB, two had TB of the spine, two had TB in lymph nodes, and one had miliary TB (Table 3). Ten cases were confirmed by culture, one by PCR, two by histology, and in two patients, it was a clinical diagnosis (based on signs and symptoms, positive interferon gamma release assay (IGRA) (QuantiFERON TB Gold), imaging, and response to TB treatment) (Table 3). Three of the patients diagnosed with TB died within the study period (129, 826, and 1,286 days after TB diagnosis, respectively).

### 3.4. Screening for Latent TB

We could obtain consistent data on screening for latent TB by an IGRA in the period from January 2010 and onwards. Tuberculin skin testing was not used to screen for latent TB in that period. A total of 4.4% of the SOT recipients and 3.7% of the patients on dialysis had been screened before SOT/start of chronic dialysis. Among patients from medium/high TB endemic areas, 16.5% of SOT recipients and 11.4% of dialysis patients had been screened, whereas among patients from low TB endemic countries, the corresponding proportions were 3.0% and 1.9%, respectively. Among 71 patients who were screened for latent TB prior to SOT or start of dialysis, 31 were from medium/high TB endemic areas and two of these patients (6.5%) developed active TB, whereas 5 (2.5%) of the 203 patients from medium/high TB endemic areas, who were not screened, were diagnosed with active TB after SOT or start of dialysis. Data on prophylactic treatment were not available.

## 4. Discussion

In this study conducted in a low TB endemic country, we found that the overall incidence of active TB among SOT and dialysis patients was 8.5 and 55 times higher than in the general population, respectively. Among study participants who were immigrants from medium/high TB endemic areas, the rates were very high, with cumulative incidences of 3-7%, whereas the incidence among patients born in countries with low TB incidence was minimal.

There are only few studies assessing the rates of active TB after SOT or dialysis in countries with low TB incidence, but our results are similar to findings from a Canadian study of SOT recipients [10], a study from the UK of patients with advanced chronic kidney disease [11], and an Australian study of dialysis patients [12]. It has previously been reported that rates of TB are 20-74 times higher in SOT recipients and patients on dialysis compared to the general population [13–15].

Patients initiating chronic dialysis tended to have a higher rate of development of active TB than patients receiving immunosuppressive therapy after SOT, but the two groups are not comparable. Dialysis patients were significantly older, and a larger proportion of dialysis patients originated from Greenland or another medium/high TB endemic country. Individuals with considerable comorbidity will not undergo SOT; thus, the SOT recipients are “generally healthier” than patients on chronic dialysis. Rates of active TB were also higher among kidney transplant recipients compared to non-kidney transplant recipients, which is likely explained by the fact that a larger proportion of kidney transplant recipients originated from medium/high TB endemic countries.

The underlying pathogenic mechanisms behind the immunosuppression induced by dialysis are not well understood. However, end-stage renal disease (ESRD) is associated with functional abnormalities in a variety of immune cells including dendritic cells, B and T cells, neutrophils, monocytes, and natural killer cells, which is not reversed by dialysis [16]. Dialysis itself may also affect immune function by causing dysfunction of neutrophils, monocytes, and T cells [17–19].

Only a small proportion of patients in the present study had been screened for latent TB prior to SOT or start of chronic dialysis. The study was not powered to determine whether screening for latent TB was associated with a reduction in risk of developing active TB after start of immunosuppressive therapy, but the efficacy of treatment of latent TB to prevent progression to active disease is well documented [20].

The sensitivity of tuberculin skin tests and IGRAs is suboptimal in immunocompromised patients [21]. In studies including immunocompetent immigrants from high TB incidence countries and TB contacts, IGRAs have exhibited a rather low ability to predict development of active TB with a positive predictive value of <1-5% [22, 23]. In these groups the number needed to treat (NNT) based on results of an IGRA to guide preventive TB treatment in low TB incidence countries has been estimated to be 30-80 [23]. The NNT may be reduced to 25 if treating only those with strongly positive test results (>4.0 IU/mL) [24]. However, estimates of positive predictive value (PPV) and NNT are sensitive to the absolute risk in the population under study, and thus, the PPV may be higher and the NNT lower among SOT recipients and dialysis patients. A modelling study estimated that the NNT based on tuberculin skin test to guide preventive TB treatment was 3-7 for patients with ESRD and a renal transplant and 6-128 for patients with ESRD requiring dialysis [25].

TB and multidrug TB treatment are associated with considerable morbidity, risk of graft failure or rejection, and substantial mortality rates [26, 27], which must be taken into consideration when determining what threshold of NNT is considered acceptable. Recent studies have showed that screening for latent TB using an IGRA prior to SOT [28] or initiation of dialysis in patients from countries with high TB prevalence [29] is cost-effective. Among patients from low TB incidence countries without a history of travel-associated, occupational, or other specific TB exposure, screening with an IGRA may not be cost-effective. Indeed, in the present study, we found very low rates of active TB after immunosuppressive therapy in patients from low TB incidence countries even in the absence of screening for latent TB.

The sensitivity of IGRAs for identifying persons who will develop active TB is suboptimal, 60-70% [22]; thus, better tools for predicting the risk of active TB are needed. Future studies of biomarkers, e.g., signature cytokine profiles of patients with protective immunity in contrast to those about to lose control of the latent infection, and progress into subclinical or clinical TB may facilitate precision medicine in management of latent TB [30].

Our study had some limitations. Data on screening for latent TB were not complete in the period before 2010, and data on prophylactic treatment for latent TB and immunosuppressive therapy for the individual patient were unavailable. The numbers of patients who developed TB were low, which is why our ability to study risk factors was limited.

Strengths of the study include the large study population with long-term follow-up and access to nationwide data and registries, which made complete ascertainment of outcomes possible. The Danish Civil Registration number ensures correct linkage of data and that patients can be followed even if they transfer to other treatment centers. Further, the registers include precise data on place of birth and data on migrations. This allowed us to calculate specific incidence rates for patients originating from medium/high TB endemic countries and for those from countries with a low incidence of TB.

## 5. Conclusion

The risk of active TB is markedly increased among SOT recipients and patients requiring chronic dialysis compared to the general population even in a country with low TB prevalence. The incidence of active TB among immunosuppressed immigrants from TB endemic areas is high, while the absolute risk of active TB is minimal among patients from low TB incidence countries if they have no history of specific TB exposure.

## Figures and Tables

**Table 1:** Characteristics of the study population, *n* (%).

	Solid organ transplant recipients (*n* = 1,989)	Patients on chronic dialysis (*n* = 1,305)
Male	1,182 (59.4)	844 (64.7)
Age, median (IQR)	48 (36-58)	64 (52-73)
Year of study inclusion		
2004-2009	763 (38.4)	603 (46.2)
2010-2017	1,226 (61.6)	702 (53.8)
Origin		
Denmark^∗^	1,756 (88.3)	1,044 (80.0)
Greenland	33 (1.7)	33 (2.5)
Other low TB endemic countries	49 (2.5)	48 (3.7)
Other medium/high TB endemic countries	151 (7.6)	180 (13.8)
SOT type		
Kidney	917 (46.1)	
Liver	530 (26.7)	
Lung	365 (18.5)	
Heart	158 (7.9)	
Pancreas-kidney	12 (0.6)	
Liver-kidney	3 (0.2)	

^∗^43 SOT and 17 dialysis patients were second-generation immigrants with parents from TB endemic countries. SOT: solid organ transplant; IQR: interquartile range; TB: tuberculosis.

**Table 2:** Incidence rates of tuberculosis stratified by the type of immune suppression and incidence rate ratios in analysis pooling of solid organ transplant recipients and patients on dialysis.

	Solid organ transplant recipients (*n* = 1,989)	Patients on chronic dialysis (*n* = 1,305)	All patients
	IR/100,000 PY (95% CI)	IR/100,000 PY (95% CI)	IRR (95% CI)^∗^
All patients	41 (15-109)	262 (145-473)	
Gender			
Male	17 (2-124)	109 (35-339)	1.0 (ref.)
Female	74 (2-230)	550 (275-1,110)	4.3 (1.4-13.7)
Age			
<50 years	17 (2-119)	364 (152-877)	1.0 (ref.)
≥50 years	78 (3-243)	212 (95-473)	2.1 (0.7-6.1)
Year of study inclusion			
2004-2009	51 (16-156)	125 (40-387)	1.0 (ref.)
2010-2017	25 (4-184)	446 (223-892)	1.8 (0.6-4.9)
Origin			
Denmark	11 (2-81)	31 (4-218)	1.0 (ref.)
Greenland	0	2,579 (645-10313)	42.2 (5.8-306)
Other low TB endemic countries	0	0	NA
Other medium/high TB endemic countries	454 (147-1,408)	1,133 (567-2,266)	49.2 (10.9-222)

IR: incidence rate; IRR: incidence rate ratio; TB: tuberculosis. ^∗^All variables were included in multivariate analyses.

**Table 3:** Characteristics of patients who developed active tuberculosis during follow-up, *n* (%).

	Solid organ transplant recipients (*n* = 4)	Patients on chronic dialysis (*n* = 11)
Male	1 (25)	3 (27)
Age < 50 years	1 (25)	5 (45)
Age ≥ 50 years	3 (75)	6 (55)
Year of study inclusion		
2004-2009	3 (75)	8 (73)
2010-2017	1 (25)	3 (27)
Origin		
Denmark	1 (25)	1 (9)
Greenland	0	2 (18)
Other non-TB endemic countries	0	0
Other TB endemic countries	3 (75)^a^	8 (73)^b^
SOT type (kidney/liver/lung/heart)	3/1/0/0	
Type of TB		
Pulmonary	4 (100)	6 (55)
Spine		2 (18)
Lymph node		2 (18)
Miliary		1 (9)
Time from SOT/dialysis to TB	2.6 years (IQR 0.6-5.3)	0.9 years (IQR 0.2-1.9)

^a^Yugoslavia: 1, Pakistan: 1, and Somalia: 1; ^b^Pakistan: 2, Philippines: 2, Rumania: 1, Somalia: 1, and Turkey: 2.

## Data Availability

Data are not publicly available.

## Additional Points

### Summary

SOT recipients and patients on dialysis had 9-55 higher rates of TB than the general population. The incidence of TB was very high in immigrants but minimal in patients from low TB endemic countries. 33% of TB cases were extrapulmonary.

## Supplementary Materials

Supplementary Materials Standard immunosuppression regimes: liver transplantation; heart transplantation; lung transplantation; and kidney transplantation.
