# Supplementary material for: Tuberculosis among Patients Undergoing Solid Organ Transplantation or Dialysis in a Low-Endemic Country, 2004-2017
Source: Tuberc Res Treat. 2020 Apr 25;2020:7636975. doi: 10.1155/2020/7636975 (PMC7201514; doi:10.1155/2020/7636975)
Supplement: Supplementary Materials — Standard immunosuppression regimes: liver transplantation; heart transplantation; lung transplantation; and kidney transplantation. [file 7636975.f1.pdf]

Standard immunosuppression regimes

Liver transplantation

Following liver transplantation, a single dose of methylprednisolone 1,000 mg is given intraoperatively. In the days following the transplantation, prednisolone is tapered gradually from 200 mg on day 1 to 30 mg on day 5. For the remaining first month, 20 mg is given daily tapered to 15, 10 and 7.5 mg daily until month 6. For the next 6 months, 5 mg is given daily after which the drug is discontinued. Tacrolimus is given twice daily, aiming at through levels of 10–12 ng/mL in the first month, 8–10 ng/mL in month 2, 7–9 ng/mL in months 3–6, 6–8 ng/mL in months 7–12 and 4–6 ng/mL after 1 year. Mycophenolate mofetil is given twice daily at a dosage of 1,000 mg continuously.

Heart transplantation

Following heart transplantation, thymoglobulin 1.5 mg/kg for 3 days with 1 g methylprednisolone for 2 days followed by 125 mg for an additional 3 days is used. Prednisone 0.2 mg/kg, tapered to 0.1 mg/kg after 3 months and to zero after 1 year, and mycophenolate mofetil 1–1.5 g × 2 daily continuously. Cyclosporine (or tacrolimus) is used adjusted to through blood levels between 200 and 350 ng/mL (10-15 ng/mL) for the first 6 weeks then reduced to 150–250 ng/mL (8-12 ng/mL) and again to 100 ng/mL (5-8 ng/mL) after one year.

Lung transplantation

Following lung transplantation, thymoglobulin 1.5 mg/kg for 3 days with 500 mg methylprednisolone for 1 day followed by 125 mg methylprednisolone each day for 3 days is used. Prednisone 15 mg daily tapered to 5 mg daily over 4 weeks. Azathioprine 1.5 mg/kg from postoperative day 1. Cyclosporine (or tacrolimus) from postoperative day 1 with target range 200–

240 ng/mL (10-15 ng/mL) for first 3 months, 150–200 ng/ml (8-12 ng/mL) for months 4–12, and again to 100-150 ng/mL (5-8 ng/mL) after one year.

Kidney transplantation

Following kidney transplantation typically, induction was given with either five doses of daclizumab or two doses of basiliximab and methylprednisolone 250-500 mg immediately before transplantation. Maintenance immunosuppression was started at day 1 as triple therapy with cyclosporine or tacrolimus, mycophenolate mofetil and steroids.
